# Maternal pre and perinatal experiences with their full-term, preterm and very preterm newborns

**DOI:** 10.1186/s12884-020-02934-8

**Published:** 2020-05-06

**Authors:** Joana L. Gonçalves, Marina Fuertes, Maria João Alves, Sandra Antunes, Ana Rita Almeida, Rute Casimiro, Margarida Santos

**Affiliations:** 1 Center for Psychology at University of Porto (CPUP), Rua Alfredo Allen, 4200-135 Porto, Portugal; 2 School of Health Technology, Polytechnic Institute of Lisbon, Lisbon, Portugal; 3 Lisbon School of Education/CIED, Polytechnic Institute of Lisbon, Lisbon, Portugal; 4 Faculty of Psychology and Education Sciences, University of Lisbon, Lisbon, Portugal

**Keywords:** Maternal representations, Prematurity, Pregnancy, Birth, Postpartum

## Abstract

**Background:**

Mothers’ reports about pregnancy, maternity and their experiences during the perinatal period have been associated with infants’ later quality of attachment and development. Yet, there has been little research with mothers of very preterm newborns. This study aimed to explore mothers’ experiences related to pregnancy, premature birth, relationship with the newborn, and future perspectives, and to compare them in the context of distinct infants’ at-birth-risk conditions.

**Methods:**

A semi-structured interview was conducted with women after birth, within the first 72 h of the newborn’s life. A total of 150 women participated and were divided in three groups: (1) 50 mothers of full-term newborns (Gestational Age (GA) ≥ 37 weeks; FT), (2) 50 mothers of preterm newborns (GA 32–36 weeks; PT) and (3) 50 mothers of very preterm newborns (GA < 32 weeks; VPT).

**Results:**

Mothers of full-term infants responded more often that their children were calm and that they did not expect difficulties in taking care of and providing for the baby. Mothers of preterm newborns although having planned and accepted well the pregnancy (with no mixed or ambivalent feelings about it) and while being optimistic about their competence to take care of the baby, mentioned feeling frightened because of the unexpected occurrence of a premature birth and its associated risks. Mothers of very preterm newborns reported more negative and distressful feelings while showing more difficulties in anticipating the experience of caring for their babies.

**Conclusion:**

The results indicate that Health Care Systems and Neonatal Care Policy should provide differentiated psychological support and responses to mothers, babies and families, taking into account the newborns’ GA and neonatal risk factors.

## Background

Generally, in the third trimester of pregnancy, women develop a clear and rich representation of their infants [1, 2], influenced by mothers’ early attachment, as well as the movements and levels of fetal activity, which are organized in cycles and patterns that are subject to interpretation by the mother [3, 4]. These maternal representations, developed during the pre- and the perinatal period, seem to affect the evolving mother-infant relationship, and prospectively to impact the quality of mother-infant interaction and later infants’ attachment security [5, 6].

Birth reveals the *“real baby”*, in contrast to the *“idealized baby”* and, as such, prenatal representations are subject to change as a result of the interactions with the *“real baby”* [7, 8]. Shortly after birth, parents form new, more accurate, objective and realistic representations of their *“real newborn”* [5]. For mothers that experience a premature birth, this adaptive process is interrupted before it is completed, and they have to nurture a fragile baby, whose survival is frequently at-risk, while simultaneously, dealing with their own feelings of fear and grief, and with their failed expectations, like “*castles in the sand”* [2, 9].

### The experience of a PT and a VPT birth

In a preterm birth, the experience of pregnancy, childbirth and early childbearing can be considerably altered due to the associated risks and danger of survival but also health risks (e.g., respiratory diseases, heart diseases, metabolic, hematological or gastrointestinal complications, difficulties in maintaining body temperature) and possible developmental sequelae (e.g., intra- and periventricular hemorrhage) [10]. Concomitantly, a premature birth may threaten parents’ expectations, idealized during the gestation, and may challenge the development of healthy or functional family dynamics [11]. Actually, the implications and the burden of coping with prematurity frequently causes in parents an emotional state characterized by feelings of great suffering and despair, unbelief and disbelief, expressed at moments of great anxiety, and grief [12]. More recently these emotions, frequently associated with maternal psychiatric conditions, such as depression, have been explored within the context of literature on parents’ post-traumatic experiences [13–16], with accumulated evidence that these symptoms persist long after the infant’s discharge from the hospital [17–23].

Indeed, mothers of premature infants exhibit higher levels of post-traumatic stress and depressive symptoms compared with controls, without recovery even 14 months after birth [17, 18, 20]. Others found that mothers with high post-traumatic stress symptoms had more distorted maternal representations affecting the interactions with their 6–month–old infants and challenging the emerging parent-infant relationship [24–26]. Contributing to parents unresolved feelings, the effect of parents’ stress, even in the prenatal period, affect intrauterine and postnatal development. For example, some studies found that maternal prenatal stress (via maternal-placental hormones) affected the fetus neurodevelopment with persisting developmental effects after birth, for instance, on infant’s behavioral reactivity in the first 3 years of postnatal life [27].

### Maternal representations and the idiosyncrasies of a PT and VPT birth

Maternal representations have been defined as mothers’ own mental and subjective representations of their experiences interacting with their infants, even before their infant’s birth, which may influence the parents’ interpretation of infant behavior and their later behavioral responses to their infant [28]. Several studies have also examined the nature and origin of maternal representations and their association with the development of attachment relationships [29].

It is well documented that mothers’ balanced and positive maternal representations of early pre- and postpartum experiences are positively associated with later secure mother-infant attachment in normative samples [30–32]. For instance, mothers who describe their infants as having a stable positive temperament are more likely to have infants who are classified as secure by the end of the first year of life [4–6, 33]. However, few studies included prematurely born infants. A longitudinal study found that, compared to mothers of full-term newborns, mothers of prematurely born infants had more negative representations of pregnancy and childbirth, and were more concerned with the health and future development of their newborn [30]. Also, according to that study, maternal representations in the first days of the newborns’ life and at 9 months postpartum were strongly associated, indicating their relative continuity and stability over time. Furthermore, attachment security at 12 months was associated with positive maternal representations at 9 months. Additionally, there is evidence that infants’ gestational age at birth has an impact on infants’ self-regulation [33, 34], quality of mother-child interaction [24, 35], quality of attachment [24, 32–36], and maternal anxiety [21, 37].

In the case of a very preterm birth, due to the extreme and traumatizing circumstances, mothers are even less likely to develop balanced maternal representations *“characterized by narratives that convey coherence, openness to change, richness of detail, and a sense of the mother as engrossed in her relationship with her infant, as they value and* *enjoy* *their relationship with their infant and are aware that this relationship affects their child’s behavior and development”* [38]. However, evidence about mothers’ initial experiences in the case of an extremely preterm birth is still very limited [39]. Available information is: (1) mainly based on case studies [40, 41]; (2) failing to compare the idiosyncrasies of mothers’ experiences in the context of different at-birth conditions [42, 43]; (3) usually focused on only one study group (e.g., very preterm infants), failing to discriminate groups of prematurity [44, 45] and (4) generally focused on parents’ reports about the prolonged time of their infants’ hospitalization, which may be different from the first early experiences in the immediate postpartum period [46, 47]. Moreover, previous evidence about maternal experience in the context of a preterm birth has presented mixed results. Some studies found that most mothers of prematurely born infants are confident about the future and optimistic about their ability to establish positive relationships with their infants [4], while other studies concluded that mothers feel alienated and have difficulty in anticipating the future [48].

### The present study

As a contribution to the body of knowledge concerning maternal representations and to the development of psychological tailored interventions to promote maternal early adjustment and later secure mother-infant relationship in the context of different neonatal birth conditions (normative, challenging or very challenging conditions), we aimed to explore prenatal and early maternal representations concerning pregnancy, birth, maternity and future perspectives. Furthermore, we aim to compare these representations among three groups: (1) mothers of full-term newborns (GA ≥ 37 weeks; FT, hereafter); (2) mothers of preterm newborns (GA between 32 and 36 weeks; PT, hereafter) and (3) mothers of very preterm newborns (GA < 32 weeks; VPT, hereafter). This study uses qualitative methods, which are best suitable to explore individuals’ idiosyncratic experiences. To our best knowledge this is the first study comparing maternal reports of pre- and postpartum experiences between these three groups.

## Methods

### Participants

A total of 150 mothers participated in this study, divided in 3 groups: (1) 50 mothers of FT newborns (GA ≥ 37 weeks, 25 girls and 25 boys), (2) 50 were mothers of PT newborns (GA between 32 and 36 weeks, 25 girls and 25 boys), and (3) 50 mothers of VPT newborns (GA < 32 weeks, 27 girls and 23 boys). Mothers’ eligibility criteria included: (1) Portuguese language fluency; (2) admission in a maternity hospital following delivery (infants born without any sensory or neuromotor disabilities, serious illnesses or congenital anomalies); (3) with no known drug/alcohol addiction problems; and (4) with no history of mental illness.

The total number of eligible mothers, in the three participating hospitals, was 177, among which 27 mothers in the preterm group refused to participate, resulting in 150 participants.

Table 1 provides descriptive statistics for infant and family socio-demographic characteristics in the three study groups.

**Table 1:** Infant and family demographics according to sample in each study group

Infant and family variables	Sample	*N*	*M*	*SD*	*Min-Max*
Apgar at first minute	FT	50	8.59	1.59	4–10
	PT	50	7.88	1.78	2–9
	VPT	50	6.55	2.38	1–9
Apgar at fifth minute	FT	50	9.83	.44	8–10
	PT	50	9.25	.87	7–10
	VPT	50	8.45	1.37	2–10
Gestational age (weeks)	FT	50	39.11	1.09	37–41
	PT	50	34.25	1.60	32–36
	VPT	50	28.25	2.47	24.5–31.6
Birthweight (g)	FT	50	3238.66	398.56	2345–4190
	PT	50	2141.25	405,837	1435–2890
	VPT	50	1165.96	453.45	500–2020
Parity	FT	50	1.33	.57	0–4
	PT	50	.80	.96	0–2
	VPT	50	1.36	.92	0–4
Maternal age	FT	50	30.32	5.12	20–43
	PT	50	27.94	5.80	18–42
	VPT	50	33.44	3.88	21–46
Maternal years of formal education	FT	50	11.24	2.25	6–19
	PT	50	10.01	3.23	4–19
	VPT	50	12.75	3.58	3–19

*Note. FT* Full-Term newborns, *PT* Preterm newborns, *VPT* Very Preterm newborns. Maternal years of education = Number of years of education that mothers completed successfully

In what concerns birth characteristics, the number of instrumental births (i.e., births with assisted delivery methods, including cesarean vs. vaginal delivery), was higher in the group of preterm infants (72%) and very preterm infants (68%). In the sample of full-term infants, the distribution of the two types of delivery was equally distributed. The occurrence of a very preterm birth was caused by: rupture of the membrane (*n* = 15), intrauterine growth restriction (*n* = 11), placental abruption (*n* = 9), early dilation (*n* = 3), preeclampsia (*n* = 2), uterine contractility (*n* = 3), baby’s bradycardia (*n* = 2), elevated urinary levels of the amino acid lysine (*n* = 2), and other non-specified causes (*n* = 3). In this group, 6 mothers used In-Vitro *Fertilization* (IVF) to conceive. The preterm births were due to: rupture of the membrane (*n* = 13), intrauterine growth restriction (*n* = 13), infection (*n* = 6), preeclampsia (*n* = 5), loss of amniotic fluid (*n* = 6), hypertension (*n* = 3), placental abruption (*n* = 3), and baby’s bradycardia (*n* = 1). Similarly, in this group, 17 mothers were enrolled in the infertility consultation to become pregnant and 6 of them used IVF to conceive. Although a small number, in the group of very premature infants, parents (*n* = 6) did not cohabit at the time of the baby’s birth (i.e., mothers were formally separated or lived apart from the infant’s father) and it was also in this group that we found a higher frequency of emigrant mothers (*n* = 12). In the full-term and in the preterm groups, the number of primiparous mothers was equally or approximately the same as the number of multiparous mothers (FT: *n* = 26 vs. *n* = 24; PT: *n* = 27 vs. *n* = 23), while in the very preterm group the number of primiparous mothers was lower (VPT: *n* = 10 vs. *n* = 40).

### Materials and procedure

The present study was conducted according to the guidelines presented in the Declaration of Helsinki, with written informed consent obtained from all individual participants included in the study (i.e., parent or legal guardian for each child), before conducting any assessment or data collection. All procedures involving human subjects in this study were approved by the Ethics Commission at the *Local Health Unit of Matosinhos*, *Francisco Xavier Hospital*, and *Hospital Center of São João*, and were developed in collaboration with the respective nursing and medical teams. All procedures regarding recruitment and data collection methods in the different hospitals were identical.

Mothers’ eligibility to participate in the study was determined through data collected from clinical files. Two female research assistants contacted personally eligible mothers after delivery and explained the study’s purpose and procedures. In this contact, their free participation was guaranteed in accordance with the American Psychological Association ethical research conduction guidelines. Eligible, consenting parents gave their written informed consent to participate. Once consent was obtained, mothers participated in an interview to collect: socio-demographic information (e.g., parental education, household, housing conditions), prenatal, perinatal and postnatal information (e.g., number of previous pregnancies, number of births, pregnancy medical follow-up, type of delivery, intercurrences at delivery, clinical status of the baby at delivery, duration of hospitalization, special care and needs, baby feeding) and post-discharge situation (e.g., baby routines, socialization, hygiene, feeding, sleep, crying, baby behavior/temperament). Through the hospital medical record, additional clinical information regarding the prenatal and perinatal conditions, of both mother and baby, relevant to the study, were obtained.

Within the first 72 h after birth, a semi-structured interview was conducted face-to-face, at a time and date that were convenient for mothers, in Portuguese maternities of three hospitals in Porto and Lisbon, where the newborns were admitted with their mothers. Interviews were audio-recorded and transcribed verbatim for qualitative analysis. The temporal window of 72 h to conduct the maternal interview was decided because: (1) it was intended to evaluate the maternal experiences in the immediate postpartum period, which is generally defined as the period between the first 48 and 96 h of postpartum hospital stay [49]; (2) in Portugal the postpartum hospitalization varies between 48 h in the case of vaginal delivery and 72 h in the case of cesarean section; and (3) this interview had been previously piloted within this time period [50].

During design, data collection, and analysis, we adhered to the consolidated criteria for reporting qualitative research (COREQ) when possible as outlined in Appendix 1 [51].

### Maternal semi-structured interview

The maternal interview, followed the procedures of a previously established protocol [50], and was used to collect mothers’ pre- and perinatal experiences in the first 72 h after infants’ birth, in the following 5 themes and 7 subthemes that resulted from a content analysis combining both grounded-theory and a hypothetical-deductive method: (1) *pregnancy* (*planned pregnancy* - planned/unexpected; pregnancy *acceptance*, e.g., father’s reaction to pregnancy announcement; other family members reaction; *support received*, e.g., family support, social support, and medical services); (2) *reaction to early birth and prematurity* (worries, fears, expectations and affectional emotions); (3) *maternal experience and relationship with the newborn child* (*feeling love for the baby for the first time*; feelings about the *first separation*); (4) *baby’s temperament* (real baby versus idealized baby); and (5) *future perspectives* (expectations and self-reflection about the *ability to care* of the baby and to engage with him/her; *expecting difficulties in the future*). The interviews were audio-recorded and lasted approximately 1 h. The validity of the interview was tested and confirmed in past research (see the interview guide in Appendix 2) [50].

### Data analyses

Bardin’s [52] method was used in the verbatim content analysis, the audio-recorded interviews were transcribed, and a content analysis was performed. Two coders carried out the content analysis of each interview independently in each sample. Then themes and subthemes were compared, and any disagreements were resolved via conferencing. When scoring was completed, the two coders compared their scores and again resolved any disagreements via conference. Overall, the average inter-coder agreement for themes and for major classification prior to conferencing in each sample was very good (80%). The results for each theme are presented in frequency for each study group. Despite this quantitative analysis, some quotes were added, particularly excerpts from the narratives of mothers of very premature infants, in order to exemplify the themes and illustrate the subthemes listed.

## Results

Given the extensiveness of our findings, in the presentation of results we will only highlight the most relevant ones (for an overview of all the themes, subthemes and the distribution of subjects in each study group among them please see the corresponding tables: Tables 2, 3, 4, 5, 6, 7, 8, 9 and 10).

**Table 2:** Frequency and percentage of maternal responses regarding pregnancy planning in each study group

Study Group	Planned Pregnancy		
	No	Yes	Mixed/Ambivalent
FT	32 (64%)	18 (36%)	0 (0%)
PT	10 (20%)	40 (80%)	0 (0%)
VPT	18 (36%)	31 (62%)	1 (2%)
Total	60 (40%)	89 (59%)	1 (1%)

*Note*. *FT* Full-Term newborns, *PT* Preterm newborns, *VPT* Very Preterm newborns

**Table 3:** Frequency and percentage of maternal responses regarding pregnancy acceptance in each study group

Study Group	Pregnancy Accepted		
	No	Yes	Mixed/Ambivalent
FT	4 (8%)	46 (92%)	0 (0%)
PT	2 (4%)	48 (96%)	0 (0%)
VPT	1 (2%)	47 (94%)	2 (4%)
Total	7 (5%)	141 (94%)	2 (1%)

*Note*. *FT* Full-Term newborns, *PT* Preterm newborns, *VPT* Very Preterm newborns

**Table 4:** Frequency and percentage of maternal responses regarding support received during pregnancy in each study group

Study Group	Support received during pregnancy		
	No	Yes	In part
FT	0 (0%)	48 (96%)	2 (4%)
PT	1 (2%)	46 (92%)	3 (6%)
VPT	7 (14%)	30 (60%)	13 (26%)
Total	8 (5%)	124 (83%)	18 (12%)

*Note*. *FT* Full-Term newborns, *PT* Preterm newborns, *VPT* Very Preterm newborns

**Table 5:** Frequency and percentage of maternal responses regarding early birth and prematurity in each study group

Study Group	Feelings of fear regarding the early birth and prematurity		
	No	Yes, felt afraid	Yes, felt so afraid that felt shock/panic
PT	15 (30%)	35 (70%)	0 (0%)
VPT	7 (14%)	19 (38%)	24 (48%)
Total	22 (22%)	54 (54%)	24 (24%)

*Note*. *PT* Preterm newborns, *VPT* Very Preterm newborns

**Table 6:** Frequency and percentage of maternal responses regarding maternal experience towards the evolving relationship with the newborn (subtheme of feeling love for the baby for the first time) in each study group

Study Group	Feelings of love for the baby for the first time						
	Does not identify	Pregnancy Confirmed	Belly Growth	When first felt the fetus movements	On the ultrasound	At birth	In the incubator
FT	1 (2%)	27 (54%)	4 (8%)	7 (14%)	4 (8%)	7 (14%)	–
PT	1 (2%)	41 (82%)	2 (4%)	1 (2%)	1 (2%)	4 (8%)	0 (0%)
VPT	3 (6%)	22 (44%)	7 (14%)	3 (6%)	2 (4%)	11 (22%)	2 (4%)
Total	5 (3%)	90 (60%)	13 (9%)	11 (7%)	7 (5%)	22 (15%)	2 (1%)

*Note. FT* Full-Term newborns, *PT* Preterm newborns, *VPT* Very Preterm newborns

**Table 7:** Frequency and percentage of maternal responses regarding first separation from the baby in each study group

Study Group	Feelings in the first separation from the baby			
	Do not know	Fear/ guilt	Felt sorry but accepted	Did not happen
FT	0 (0%)	2 (4%)	0 (0%)	48 (96%)
PT	3 (6%)	28 (56%)	16 (32%)	3 (6%)
VPT	3 (6%)	17 (34%)	30 (60%)	0 (0%)
Total	6 (4%)	47 (31%)	46 (31%)	51 (34%)

*Note*. *FT* Full-Term newborns, *PT* Preterm newborns, *VPT* Very Preterm newborns

**Table 8:** Frequency and percentage of maternal descriptions of newborn’s temperament in each study group

Study Group	Characterization of newborn*’*s temperament			
	Do not know	Calm	Agitated/ Weeping	Mixed
FT	0 (0%)	33 (66%)	10 (20%)	7 (14%)
PT	10 (20%)	30 (60%)	5 (10%)	5 (10%)
VPT	16 (32%)	13 (26%)	20 (40%)	1 (2%)
Total	26 (17%)	76 (51%)	35 (23%)	13 (9%)

*Note*. *FT* Full-Term newborns, *PT* Preterm newborns, *VPT* Very Preterm newborns

**Table 9:** Frequency and percentage of maternal responses regarding the future perspectives (ability to take care of and to rear their baby) in each study group

Study Group	Taking care of the baby in the future				
	Do not know	Good and it will go well	Difficult but will learn	Afraid of not knowing	Do not want to think about it now
FT	7 (14%)	30 (60%)	1 (2%)	12 (24%)	0 (0%)
PT	3 (6%)	38 (76%)	5 (10%)	4 (8%)	0 (0%)
VPT	6 (12%)	28 (56%)	11 (22%)	1 (2%)	4 (8%)
Total	16 (11%)	96 (64%)	17 (11%)	17 (11%)	4 (3%)

*Note. FT* Full-Term newborns, *PT* Preterm newborns, *VPT* Very Preterm newborns

**Table 10:** Frequency and percentage of maternal responses regarding the subtheme of expecting difficulties in the future in each study group

Study Group	Difficulties in the future				
	Do not know	None	Some but will ask for help	Anticipates many difficulties	Do not want to think aboutit now
FT	0 (0%)	15 (30%)	30 (60%)	5 (10%)	0 (0%)
PT	8 (16%)	23 (46%)	8 (16%)	11 (22%)	0 (0%)
VPT	18 (36%)	10 (20%)	10 (20%)	1 (2%)	11 (22%)
Total	26 (18%)	48 (32%)	48 (32%)	17 (11%)	11 (7%)

*Note. FT* Full-Term newborns, *PT* Preterm newborns, *VPT* Very Preterm newborns

Overall, five main themes were discerned in the data: (1) pregnancy; (2) early birth and prematurity; (3) maternal experience and the relationship with the newborn; (4) baby*’*s temperament; and (5) future perspectives. Several subthemes, discussed below, emerged in the data further describing how mothers viewed both their pre- and perinatal experiences.

### Theme 1: pregnancy

Regarding the first theme of *pregnancy* and the subtheme of *planned pregnancy*, most mothers of PT infants (80%) and VPT infants (62%) planned their pregnancy, in contrast with 36% in the group of FT mothers (see Table 2).

In the *pregnancy acceptance* subtheme, most mothers accepted pregnancy well (94%). Non-acceptance of pregnancy was found in 8% of FT mothers, 4% of mothers of PT infants and 2% of mothers of VPT infants. Moreover, 4% of mothers of VPT infants reported feeling ambivalent about their pregnancy: *“I did not want a baby, I was on a diet. My eldest son is fourteen, and now I wanted to have my own life. My husband was so happy that it irritated me. It was only when I started reviewing my son’s baby pictures, that I started liking her”* (M70) (for more details see Table 3).

Regarding the subtheme *support received*, all mothers of FT infants felt partially (4%) or totally (96%) supported during pregnancy, both by health professionals and/or by closed ones such as family/friends. Likewise, 92% of mothers of PT infants felt fully supported and only one mother said she felt she had no support (2%). As for the group of mothers of VPT infants, 60% reported feeling totally supported, while 14% mentioned not having received any support. For instance, one mother noted: “*The pregnancy was complicated. I was very nervous; my husband was arrested. I was alone and pregnant; I had another child and I still had to work because there was no one who could help me financially and someone had to pay the bills”* (M21) (see Table 4).

### Theme 2: early birth and prematurity

As for the second theme of *reaction to the early birth and prematurity* (see Table 5), the results show that 70% of mothers of PT newborns feared for their baby’s life. In the group of mothers of VPT newborns, 86% revealed feeling fear, and between them 48% characterized their birth experience with feelings of shock/panic that were both paralyzing and devastating. In their words: “*I was sobbing so hard I couldn’t catch my breath; the panic was so overwhelming. It was a shock, I started trembling and shaking, I had no signs of labor, nor pains, and suddenly the waters broke”* (M27). Interestingly, 30% of mothers of PT newborns and 14% of mothers of VPT newborns said that they were not afraid for the baby’s life. As an example: “*They (medical doctors) reassured me that there are a lot of babies like him that turn out okay. I believe in them because I have cousins ​​who were born premature who are now big and strong adults”* (M46).

### Theme 3: maternal experience and the relationship with the newborn

In the third theme of *maternal experience and relationship with the newborn child*, and in the subtheme *feeling love for the baby for the first time* (see Table 6), most mothers reported that they were first aware of this feeling at a very early stage, when pregnancy was confirmed, which occurred in 54% of mothers of FT infants, 82% of mothers of PT infants and 44% of mothers of VPT infants. This feeling was described, for instance, as follows: “*I felt love since the moment I knew I was pregnant … there was a human being growing inside of me, the fruit of our love”* (M24). Other mothers only described the emergence of this feeling later, namely, at birth (14% of mothers of FT babies and 22% of mothers of VPT babies), when they saw the *“real”* baby, as one mother explained: “*As soon as I saw him it was clear to me how much I loved him!”* (M19).

In the subtheme of *first separation from the baby* (see Table 7), most mothers of FT infants (96%) reported not having been separated from their infants after birth, while virtually all mothers of PT infants had the opposite experience. As a reaction to the first separation, more than half of the mothers of PT infants described their experience with feelings of great pain, fear and/or guilt (56%). Most mothers (60%) of VPT babies outlined the experience of having mixed feelings of both sorrow and acceptance, for example, as expressed by one mother: “*It is always painful, but my daughter is having everything she needs to grow in the outside world”* (M31). In the same group, this feeling was followed by detailed descriptions that revealed a lot of suffering and pain as well as the emergence of feelings of fear/guilt (34%), as in the following case: “*My first thought was: I did something wrong, I failed! I felt ashamed and scared”* (M60). In both groups (FT and VPT), three mothers did not know how to describe their experience or *“how to translate into words”* (M14) what they felt when they were first separated from their babies (6%): “*I do not know what to say … sure I wanted to go with my baby, but I could not”* (M14).

### Theme 4: baby*’*s temperament

In the fourth theme of *baby’s temperament* (see Table 8) most mothers of FT infants (66%) characterized their baby’s temperament using the adjective *“calm”*. Similarly, most mothers of PT babies described them as calm (60%), as in *“she is very peaceful, silent and quite, observant and attentive … she is so calm that sometimes I think that she believes she is still in my belly … she is loving, sometimes I cuddle her in the nose and she smiles. I thought that she would be bossy because she was electric while on my belly, she moved a lot during the pregnancy but actually she is a doll and I just feel like cuddling her”* (M22), while 10% considered their baby to be agitated and constantly weeping, pointing for instance*, “she has quite a temper! She has a strong personality and she only does what she feels she wants to do … she is also very nervous, agitated … she is a difficult baby, very demanding, she cries a lot and does not sleep well, she can’t even stop at night, at least she could let me sleep so that I could rest in order to meet her needs during the day, she does not make things easier”* (M15). The responses of mothers of VPT babies were more diverse, fluctuating between agitated/weeping (40%), calm (26%), and not knowing how to describe them (32%).

### Theme 5: future perspectives

In the fifth theme of *future perspectives* (see Table 9), in the subtheme of *take care of their baby* (Table 9), 60% of mothers of FT babies, 76% of mothers of PT babies and 56% of mothers of VPT babies said that they anticipated that everything would go well when thinking about their future caring abilities: “*In a way, it’s going to be better than it was with my other child because now it’s not the first [maternity] experience”* (M57); “*It will be very good, to finally take care of her, not being dependent on any medical equipment”* (M34). Contrary, some mothers (22% of VPT babies, 10% of PT babies, and 2% of FT babies) mentioned that it would be difficult, but that they were willing to learn.

Regarding the subtheme of *expecting difficulties in the future* (see Table 10), when asked to imagine the potential struggles and/or complications they would face prospectively after discharge, most mothers of FT babies considered that they would have difficulties although, in that case, they would ask for help, which they thought would be available and responsive (60%), and about a third were confident that they would have no difficulties (30%). Almost half of the mothers of PT infants considered that they would have no difficulties (46%) and about a quarter considered they could experience several difficulties (22%). Finally, in the group of VPT infants, mothers expressed and/or revealed difficulties in anticipating the future. Actually, 36% of mothers in this group roughly admitted that they did not know how to answer the question, when, for instance, one mother said: “*I have no idea what the difficulty will be, but to leave all this behind … the team of nurses and doctors, always available to help, without any hesitation, to clarify any doubt and to assist in case of need and the medical equipment, which is always here and available for any emergency, and to imagine that it will be just the two of us...well, I do not know how it will be”* (M1). Beyond the difficulty revealed in the process of anticipating the future, 22% of these mothers preferred not to think about it: “*I have started to think about it, but I do not want to think about it too much because those kind of thoughts do not help me and I feel like I have to focus on the present moment and live day-by-day, one day at a time”* (M31).

## Discussion

In this study, we explored maternal early experiences related to pregnancy, early birth, maternity and the relationship with the newborn, and future perspectives regarding parenting. Furthermore, we compared them in the context of distinct infants’ at-birth-risk conditions, by including in our sample mothers of full-term, preterm, and very preterm newborns. A semi-structured interview was conducted 72 h after delivery and results allowed to distinguish both similarities and differences among these groups.

### Mothers of full-term infants

In the group of *full-term (FT) mothers*, most mothers did not plan their pregnancy, but the majority mentioned they had accepted it. The larger part of mothers reported to have felt love for their baby when they found they were expecting. Almost all mothers felt supported by both health professionals and/or family/friends during gestation. During their hospital stay, most mothers described their baby’s temperament as calm and only about a quarter described them as restless or difficult. Anticipating the future, these mothers felt that it would be good to take care of their babies, but the majority expected to find difficulties to be overcome with the help from family support (which they anticipated having).

### Mothers of preterm infants

Most *mothers of preterm (PT) babies* planned and accepted well their pregnancies. In fact, a large part of mothers in this group was enrolled in the infertility medical consultation (assisted reproduction) to become pregnant. These mothers reported having felt love for their child as soon as they learned they were pregnant. Despite these positive aspects, when birth occurred prematurely, mothers felt concern or great concern and guilt. As in previous studies, mothers of PT infants described feelings of distress and despair regarding the experience of pregnancy and birth [39]. However, as found in the study of Fuertes et al. [30] with mothers of prematurely born infants (born between 26 and 32 weeks of gestation), the future was anticipated with optimism by most mothers. Compared with the other two groups, fewer mothers of premature babies expected difficulties and more mothers believed that it would be good to take care of their newborns after discharge. Nevertheless, similar previous studies [48, 53] reported that preterm mothers’ experiences were punctuated by ambivalent, mixed feelings, oscillating between the optimism toward the future and the distress and fear resulting from the unexpected preterm delivery and their babies’ frequently unstable and unpredictable clinical condition.

### Mothers of very preterm infants

The most striking results of this study concern the narratives of *mothers of* *very preterm (VPT) infants*. The pregnancy and early postpartum experience reported by *mothers of very preterm infants* is equally accepted, but compared to the other two groups, the feeling of love for the newborn occurs much less frequently at an early stage, for instance, when mothers first are informed about their pregnancy. Worryingly, in this group, the first feeling of love is described as occurring only after birth by about one-third of the mothers and, even more serious, three mothers did not express this affection for their baby at all. Regarding this dimension it is important to note that we did not present mothers with any definition of the expression *“feeling love”*. In fact, some mothers expressed having doubts about the meaning of *“feeling love”* and were not sure about having ever felt that sensation while others defined *“feeling love”* as the *“urgency”* of being close, to care, to hold, to love, to protect and as a complex, confusing and to some extent scary but positive and strong bonding feeling. To our best knowledge, no previous study found differences between the three groups of mothers concerning the moment when these feelings arouse for the first time.

Furthermore, since several studies found that the prevalence of depression is higher in mothers of very or extremely preterm infants [54], we wonder about a possible association between maternal mental health conditions and infant birth status.

Actually, not only did the life scripts of these mothers carry more risk indicators (e.g., there were more emigrant mothers, mothers separated from the infant’s fathers) but, also, they reported more cumulative risk situations at that present moment. For example, some of them had not received any personal support during pregnancy or clinical/health follow-up, which is rare in the Portuguese context [55]. These mothers described the birth of their baby as a traumatic, panic-inducing experience, although showing simultaneously some level of emotional regulation through adaptive coping strategies and improved cognitive flexibility by accepting the process of separation from their baby so that he or she could receive the required special care. Other studies found similar results [56]. Nonetheless, the distinction between representations of PT and VPT mothers is much clearer in our study: whereas mothers of PT newborns reported feelings of distress, worry and concern, mothers of VPT newborns used expressions like panic or trauma to characterize their experiences.

When asked to describe their babies’ temperament a large group of mothers of VPT infants reported perceiving their infant as agitated, and a significant number of mothers showed difficulties in characterizing the baby’s temperament. These results may be explained by both (1) the mothers’ emotions and their main focus on their newborn struggle for survival in the immediate moment which prevent them from being sensitive to other characteristics of their baby; and (2) by the physical immaturity of the very preterm newborn which, as reported in other studies, results in greater difficulty for parent-newborn interaction [57].

When asked to reflect upon their perceptions and expectations for the future, mothers of VPT infants were so focused on their newborn struggle for survival in the immediate moment that they showed reluctance in looking into the future. Again, these findings may be due to the frightening uncertainty about their baby’s survival in this phase. Uncertainty and grief usually prevent mothers from being able to perceive and to reflect about potential difficulties in the future.

Lastly, although it was not defined in our initial research aims, given the high and unexpected number of births by caesarean section, we decided to analyze the impact of this variable on our findings. Indeed, the type of delivery affected mother’s perception of the baby’s temperament (mothers who gave birth by caesarean section described more often their newborns as agitated) as well as the anticipation of prospective problems or potential future difficulties (see Appendix 3). Like in previous studies, mothers who gave birth by caesarean section, had more negative representations of their perceived control, emotions, and the quality of first moments with the newborn compared to vaginal birth [58].

### Summary of findings, general reflections and clinical implications

In summary, mothers’ early perception of their experiences with full-term, preterm and very preterm infants showed some similarities but equally important differences. These results reinforce previous evidence indicating that maternal experiences in these three groups are distinct [43], especially when the infant is hospitalized and/or stays at the Neonatal Intensive Care Unit (NICU) and needs adequate and specific clinical intervention and medical care [10].

In fact, the growing recognition that parents’ negative emotions (e.g., anxiety, depression) and stress are associated not only with prematurity but also with the quality of their experience during their stay at the NICU contributed to the significant enhancement of Family-Centered Care (FCC) within NICU and to the development of family-centered interventions during NICU hospitalization [59, 60]. These interventions have been tailored to meet parents needs and to the promotion of parental and baby interaction, parental involvement and parental sense of self-efficacy in the care of their babies [61, 62]. Our results reinforce the relevance of these interventions and highlight the need for further development of intervention programs, strategies and policies in support of these babies and their parents. Our findings concerning mothers’ feelings (e.g., fear, panic and guilt) and the evidence suggesting an association between maternal reports of pre- and perinatal experiences and the quality of mother-infant attachment [30, 32], point to the importance of including well-trained professionals in attachment intervention programs to help mothers cope with these stressful experiences and to develop more positive representations, to engage in more positive interactions as well as to enhance first bonding experiences with their newborns. The differences that we found among mothers experiences (in the case of a full–term, preterm and very preterm birth) need to be addressed in providing care for these families. Moreover, evidence suggests that the first experiences of contact with the world are especially relevant for infant’s later socio-emotional development, namely it has been found that early social experience affects the developing brain and thereby infants social, cognitive, and emotional development [63]. Infants’ early postnatal period is characterized by heightened brain plasticity and, in particular, high sensitivity to social environmental influences [64]. Such social influences comprise a constellation of context and caretaking features – including face-to-face interactions and tactile contact – that are critical for optimal child cognitive and socioemotional development [65, 66]. For instance, infant studies have shown that key components of the neural network for socioemotional processing (e.g., amygdala, temporal, and frontal cortex) operate from very early in postnatal life, at the time when perceptual representation areas are attuned to relevant social signals, including faces, direct gaze, facial expressions, and social interaction contingencies [67]. Bearing in mind the aforementioned role of infants’ early experience on their sensitivity to social relevant signals (e.g., facial expressions), although much has been done to promote parent-newborn proximity in the NICU, this environment still limits mothers’ opportunities to establish intimate contact with their newborn. This aspect reinforces the urgent need for more early on intervention programs in the context of this particular birth condition, as there is evidence that if precocious supportive intervention, namely with a preventive approach, is provided for infants and their families, many children recover from their difficult start [68–73].

In our study, mothers of very preterm newborns showed more negative emotions and more difficulty in facing the future. The fragile clinical conditions of very preterm infants, the incidence of mortality among these babies and the extremely stressful conditions of anxiety and depression may prevent mothers from engaging with their baby [74]. These symptoms may affect maternal feelings and condition their responses, which are crucial for their interactions and the evolving mother-infant relationship [75]. Importantly, several studies have indicated that the quality of mother–infant interaction differs in dyads with preterm or very preterm infants [76–79]. Comparative studies of full-term infants, low-risk preterm infants and high-risk preterm infants indicate that very preterm infants are significantly more limited in sustained attention and show higher negative affect during social interactions than the other two groups, whose facial expressions are frequently more ambiguous and difficult to interpret and thus, challenging mother-infant bonding [77, 80, 81]. The mothers of these infants tend to initiate interactions without clear cues from their infants, or interactions that are less clearly adjusted to infant cues. They appear to express less positive affect, and to experience less pleasure in interacting with their babies. Some authors have also characterized them as tending to be more overstimulating or less involved in the interaction than other mothers [24, 25, 35, 82–85].

Furthermore, mothers of VPT infants face a long stay in the NICU which frequently has repercussions in their personal and family lives. Their specific needs pose new challenges that must be addressed in the NICU, and more generally by the Health Care Systems and Neonatal Care Policy, including government and global maternal and child health care agencies.

### Strengths and limitations

Our study has both strengths and limitations. The current study was developed within a descriptive and interpretative paradigm, using a qualitative methodology, without generalization concerns. Indeed, one of our limitations it that our results mainly mirror the reality of Portuguese mothers living in urban areas. However, it is noteworthy that our study includes a large sample of 150 mother-infant dyads which, although distributed across three study groups, allows us to deepen our data exploration and the uniqueness of each group experience.

Importantly, in our sample, the three study groups are distinct from the point of view of family conditions, history of pregnancy and attempts to conceive (e.g., planning and acceptance of pregnancy), which should be considered when reading our results. In fact, in addition to the risk-factors associated with a preterm birth, the group of very premature infants presents more risk-factors associated with pregnancy and family relationships (support network), compared with the other two groups, which may increase mothers’ sense of insecurity, influencing the nature of their postpartum experiences. Future studies must address questions of diversity, marginalization, and interlocking oppressions in this particular group.

Mothers experience in each group, also, varied regarding breastfeeding experiences (none of the mothers of very preterm newborns breastfed their baby before the interview while all mothers of FT infants did so) and labor (caesarean deliveries were more prevalent in the VPT sample). Although the samples vary in these aspects, in fact, they reflect the different experience in these groups. The unexpected and high number of caesarean sections deserves further investigation, namely regarding its causes and consequences.

To learn more about the participants’ perspectives, we invited a group of 10 parents (both mothers and fathers) in the preterm group to participate in a videotaped focus group to discuss and reflect about the study findings. The parents confirmed that the identified themes and subthemes reflected their experience, demonstrating an identification with the study results and stressing the need for more effective support responses in the hospital maternity and after discharge. Further research addressing the temporal continuity of these early experiences, namely the impact of parental stress and early relationships on infants’ development, is pivotal. For instance, although it is generally accepted that parental stress is influenced by infants’ gestational age and weight at-birth, and particularly with pre- and perinatal complications [22], some parents remain fearful even in the absence of any health or developmental complications [86, 87]. Moreover, in our future studies, we aim to include mothers from different backgrounds [88], as well as the fathers’ perspective [89].

At last, it is important to stress that preterm birth is the most common single cause of perinatal and infant mortality, affecting 15 million infants worldwide each year with global rates increasing exponentially [90]. In recent studies, a *“call to action”* has been proposed highlighting the urgent need to understand the etiology and the context of a preterm birth (e.g., temporal trends, patterns, predictors, and outcomes), focusing on the need to use new strategies, namely prevention strategies (e.g., going beyond progesterone) to tackle the combined biological and social factors associated with an early birth [90–97]. For instance, a cross-country study analyzed 4.1 million singleton births in 5 countries with very high human development index and found no biologic explanation for 2/3 of all preterm births, underlining the need to consider other risk factors, such as diet, stress, periodontal disease, and other maternal or fetal clinical risk factors, in order to develop more effective preventive interventions [90].

## Conclusion

We believe that this study contributes to the body of knowledge that describes maternal pre- and perinatal experiences with their newborns in the context of different neonatal risk factors. The exploration and understanding of early life subjective experiences and their causes are fundamental in enhancing successful evidence-based mother-infant intervention strategies and medical-clinical practice, while simultaneously informing theoretical, practical, research, policy-making and public health decisions.

## Data Availability

Non applicable, given that the raw data would potential identify participants it is not deemed appropriate to share.

## Appendix 1

**Table 11:** COREQ Checklist: Methods and reporting according to COREQ statement

No Item	Description
**Domain 1: Research team and reflexivity**	
**Personal Characteristics**	
1. Interviewer	JLG, MF, and MJA conducted the interviews.
2. Credentials	Three psychologists conducted the interviews: JLG, MSc in Psychology MF, PhD in Psychology MJA, MSc in Psychology
3. Occupation	JLG was a PhD student at the University of Porto. MF was an Associate Professor at School of Education, Lisbon Polytechnic Institute and a Researcher at the Centre for Psychology, University of Porto. MJA was a researcher at School of Education, Lisbon Polytechnic Institute. JLG was awarded with the cited doctoral grant from the Portuguese Foundation for Science and Technology (SFRH/BD/90853/2012). MF was the PI of the project funded by the cited grant from the Portuguese Foundation for Science and Technology/FEDER (PTDC/MHC-PED/1424/2014). MJA received salary support from the cited grant from the Portuguese Foundation for Science and Technology/FEDER (PTDC/MHC-PED/1424/2014).
4. Gender	All interviewers were female.
5. Experience and training	All interviewers had prior interviewing experience. Qualitative research training in interview conduction and analysis was provided to JLG, MF and MJA within the scope of their academic training in Psychology.
**Relationship with participants**	
6. Relationship established	A prior relationship between the interviewer and participants did not exist (with the exception of the brief initial contact at the time of recruitment, when mothers were invited to participate in the study).
7. Participant knowledge of the interviewer	Prior to any data collection, we provided all potential participants with an overview of the study prior to signing of the informed consent. The interviewer provided a brief introduction prior to beginning an interview.
8. Interviewer characteristics	No specific bias or assumptions to declare for any of the interviewers. All interviewers have been working on the field of clinical, neonatal and developmental psychology, with relevant published work concerning mother-infant relationship in the context of distinct infants’ at-birth-risk conditions, with a particular focus on the preterm birth within the scope of their research interests.
**Domain 2: Study design**	
**Theoretical framework**	
9. Methodological orientation and Theory	The maternal interview followed the procedures of a previously established protocol [50], to collect mothers’ pre- and perinatal experiences in the first 72 h after infants’ birth. Following the guidelines used in the previous study, we used content analysis, combining both grounded-theory and a hypothetical-deductive method which resulted in 5 themes and 7 subthemes. Using grounded theory to inductively derive additional themes that characterized the transcripts allowed to further describe our findings.
10. Sampling	Purposive sampling.
11. Method of approach	Participants were identified by two female research assistants who contacted personally, face-to-face, eligible mothers after delivery and explained the study’s purpose and procedures.
12. Sample size	Although the total sample size in the study is large, there are three sub-groups, allowing in-depth analysis of all viewpoints. The three groups of mothers were recruited, with the aim of exploring their maternal pre- and perinatal experiences in depth, in the context of distinct infants’ at-birth-risk conditions. Content analysis does not preclude the use of large numbers and, as with all qualitative research, does not claim generalisations [98]. According to Bengtsson [99], in qualitative studies, normally, data are based on 1 to 30 participants. However, the author stresses that sample size should be determined on the basis of informational needs so that it is possible to organize and elicit meaning from the data collected and to draw realistic conclusions from it, and ultimately, to answer to the research question at study with sufficient confidence. In our study, we expected a large diversity of responses and speeches (considering the large diversity of mothers’ backgrounds and health conditions associated with prematurity) resulting in several indicators in each *unit of analysis*. Therefore, we decided to enlarge the sample to 50 participants to have enough representative and clarifying information for each subtheme and indicator in each group.
13. Non-participation	No mother withdrew from participating in our study after signing the informed consent.
**Setting**	
14. Setting of data collection	At each of the three participating hospitals, we asked for a quiet silent room or office available in the maternity or nearby where the interview was conducted.
15. Presence of non-participants	Our protocol did not allow non-participants. Any non-participants were immediately asked to leave if they entered the room where an interview took place.
16. Description of sample	Our protocol allowed for collection of demographics, family, socio-economic characteristics, as well as medical and clinical information of the infant, mother and their families.
**Data collection**	
17. Interview guide	This was pilot tested in a previous study [50]. The interview protocol is available as additional file in Appendix 2.
18. Repeat interviews	No, our protocol was defined so that the interview should be conducted only one time, at the first 72 h postpartum, although flexibility was given for mothers to choose the best and most appropriate time within this temporal window. It was not our aim to conduct the same interview with the same participant more than one time.
19. Audio/visual recording	Digital audio recordings were made and transcribed verbatim.
20. Field notes	Field notes were not taken since the digital recordings were rapidly reviewed upon the return of the study team to the research center at the University.
21. Duration	The individual one-on-one and face-to-face interviews lasted approximately 1 h.
22. Data saturation	Lincoln and Guba’s [100] four criteria for trustworthiness (credibility, dependability, confirmability and transferability) were used to ensure rigor in the qualitative process. Notes were taken while data coding to capture the participants’ voices and the meaning behind their words and to assist in eliminating potential bias or misinterpretation. Data analysis began with the first interview and continued through formulation of the five main themes, the various subthemes and into reporting of the findings. In qualitative research, the data lend itself to interpretation by the researcher. The themes extrapolated from the data were supported with rich data and use of participant’s own words added to the authenticity. Data saturation was reached early in the process; however, the researcher continued to read and reread each transcript with attention to detail. The lived experiences of the participants were revealed and not subject to bias of the researcher.
23. Transcripts returned	We did not return transcripts to participants for comment and/or correction. However, (1) participants were provided with information related to dissemination of the research findings and given time to ask questions related to the basis for the research and (2) a group of 10 parents (both mothers and fathers) in the preterm group were invited to take part in a videotaped focus group to discuss and reflect about the study findings. Regarding ethical considerations, the transcripts had personal identifiers that were removed to protect the participants in the event of a security breach. After reviewing the transcripts for errors and inserting field notes, the researcher deleted all audio recordings.
**Domain 3: Analysis and findings**	
**Data analysis**	
24. Number of data coders	All the seven authors participated in the coding process.
25. Description of the coding tree	The coding tree used for initial assignment into the 5 major themes, that had already been used in a previous study [50], is described in the methods.
26. Derivation of themes	Major themes were derived in advance; minor or subthemes were derived from the data inductively.
27. Software	No software was used.
28. Participant checking	To learn more about the participants’ perspectives, we invited a group of 10 parents (both mothers and fathers) in the preterm group to take part in a videotaped focus group to discuss and reflect about the study findings. The parents confirmed that the identified themes and subthemes reflected their experience, demonstrating an identification with the study results and stressing the need for more effective support responses in the hospital maternity and after discharge.
**Reporting**	
29. Quotations presented	See results section (participants quotations were presented to illustrate the findings and each quotation is identified through the participant number).
30. Data and findings consistent	Questions 30–32 of COREQ address the evaluation of the findings of a qualitative study and are intended for readers of qualitative research. They are included here for completeness. We have attempted to present our findings in this work clearly in a manner that was consistent with the data collected.
31. Clarity of major themes	Major themes were clearly presented and discussed in the results and discussion sections.
32. Clarity of minor themes	Minor themes were clearly presented and discussed in the results and discussion sections.

## Appendix 2

### THE MATERNAL INTERVIEW [50]

PREGNANCY

1. Do you remember the moment when you knew you were pregnant? How did you feel (planned/unexpected pregnancy/desired)?

2. How was the reaction of your husband/partner? And how did your family react?

3. Did you feel supported during pregnancy (by friends, partner, family, and health professionals)?

EMOTIONS CONCERNING PREGNANCY AND LABOR

4. Do you remember what you felt the first time your baby moved inside of you?

5. As your baby grew inside of you, how did you imagine him/her?

6. Were you afraid that something might happen to your baby? How was the labor?

FIRST 2 DAYS OF EXPERIENCE

7. Remember the moment you first felt love for your baby?

8. What did you feel when you first held your baby?

9. How easy/difficult it is to take care of him or her?

10. How would you describe your baby’s temperament?

FUTURE PERSPECTIVES

11. What will it be like to take care of your child at home? How do you expect to relate with him/her?

12. Do you expect some difficulties?

13. How do you imagine your baby’s future (health and development)?

## Appendix 3

Although the research hereby presented did not aim to address differences in maternal experiences according to type of delivery, we found differences between maternal representations in vaginal vs. caesarean delivery. Mothers with caesarean delivery described more frequently their babies as agitated (see Table 12) and presented more difficulties in anticipating possible difficulties in future baby care (see Table 13).

**Table 12:** Frequency and percentage of maternal descriptions of newborn’s temperament according to type of labor

		Characterization of newborn*’*s temperament			
		Do not know	Calm	Agitated/Weeping	Mixed
Birth	Vaginal	13 (22,8%)	28 (49,1%)	11 (19,3%)	5 (8,8%)
	Caesarean	23 (20,5%)	47 (42,0%)	35 (31,3%)	7 (6,3%)
	Total	36 (21,3%)	75 (44,4%)	46 (27,2%)	12 (7,1%)

**Table 13:** Frequency and percentage of maternal responses regarding the subtheme of difficulties in the future according to type of labor

		Difficulties in the future				
		Do not know	None	Some but will ask for help	Anticipates many difficulties	Do not want to think about it now
Birth	Vaginal	5 (8,6%)	16 (27,6%)	25 (43,1%)	5 (8,6%)	7 (12,1%)
	Caesarean	23 (20,2%)	38 (33,3%)	32 (28,1%)	12 (10,5%)	9 (7,9%)
	Total	28 (16,3%)	54 (31,4%)	57 (33,1%)	17 (9,9%)	16 (9,3%)

## Abbreviations

COREQ: COnsolidated criteria for REporting Qualitative research
FCC: Family-Centered Care
FT: Full-Term
GA: Gestational Age
IVF: In-Vitro Fertilization
NICU: Neonatal Intensive Care Unit
PT: Preterm
VPT: Very Preterm
